# Lactate Shuttles in Neuroenergetics—Homeostasis, Allostasis and Beyond

**DOI:** 10.3389/fnins.2017.00043

**Published:** 2017-02-02

**Authors:** Shayne Mason

**Affiliations:** Centre for Human Metabolomics, North-West UniversityPotchefstroom, South Africa

**Keywords:** neuroenergetics, lactate, astrocyte-neuron lactate shuttle (ANLS), astrocyte-microglia lactate shuttle (AMLS), neuropathology, traumatic brain injury (TBI), neurodegenerative disease, infectious neuroinflammatory disease

## Abstract

Understanding brain energy metabolism—neuroenergetics—is becoming increasingly important as it can be identified repeatedly as the source of neurological perturbations. Within the scientific community we are seeing a shift in paradigms from the traditional neurocentric view to that of a more dynamic, integrated one where astrocytes are no longer considered as being just supportive, and activated microglia have a profound influence. Lactate is emerging as the “good guy,” contrasting its classical “bad guy” position in the now superseded medical literature. This review begins with the evolution of the concept of “lactate shuttles”; goes on to the recent shift in ideas regarding normal neuroenergetics (homeostasis)—specifically, the astrocyte–neuron lactate shuttle; and progresses to covering the metabolic implications whereby homeostasis is lost—a state of allostasis, and the function of microglia. The role of lactate, as a substrate and shuttle, is reviewed in light of allostatic stress, and beyond—in an acute state of allostatic stress in terms of physical brain trauma, and reflected upon with respect to persistent stress as allostatic overload—neurodegenerative diseases. Finally, the recently proposed astrocyte–microglia lactate shuttle is discussed in terms of chronic neuroinflammatory infectious diseases, using tuberculous meningitis as an example. The novelty extended by this review is that the directionality of lactate, as shuttles in the brain, in neuropathophysiological states is emerging as crucial in neuroenergetics.

## Introduction

The human brain represents approximately 2% of total body weight and receives up to 15% of total blood flow, consuming up to 20% of oxygen and 25% of circulating glucose under normal conditions (Pellerin, 2010). The metabolism of this high energy consuming organ involves complex intercellular trafficking of metabolites and compartmentalization of numerous processes. Tight coupling exists between the supply and demand of energy in brain metabolism with changes in cerebral blood flow and glucose utilization in response to neuronal activity (i.e., neurovascular and neurometabolic coupling) (Bélanger et al., 2011) under normal homeostatic conditions. Mechanisms involved in brain energy metabolism adapt during periods of perturbation and trauma. The important role of lactate, and its shuttles, has been overlooked and merits acknowledgement.

The interconversion of lactate and pyruvate occurs via lactate dehydrogenase, with increased lactate typically being associated with anaerobic respiration. Thus, one could postulate that elevated levels of lactate present in the cerebrospinal fluid (CSF) due to neuroinflammation could be from hypoxia caused by ischemia—increased anaerobic respiration (Rossi et al., 2007), or by raised glucose levels and hence elevated flow through the glycolysis pathway. However, in most neuroinflammatory cases there are typically periods of low levels of glucose in the CSF. Furthermore, several studies have shown no correlation between CSF lactate levels and cerebral blood flow (i.e., they are unrelated to ischemia) (Brodersen and Jorgensen, 1974; DeSalles et al., 1986; De Salles et al., 1987). Thus, an alternative postulate is that the elevated levels of lactate in the CSF of neuroinflammatory cases is unlikely to be due to anaerobic respiration but instead is possibly a product of temporarily increased flux in the glycolysis pathway—using glycogen as a supplementary source of glucose.

The notion that lactate is not simply just a by-product of glycolysis but also a shuttle system was pioneered by Brooks (1985). In June the following year, Brooks provided more evidence for a “shuttling” system for lactate (Brooks, 1986a); by December Brooks (1986b) formally coined the term “lactate shuttle” in skeletal muscle during rest and exercise under fully aerobic conditions. Brooks further defined lactate shuttles as being either intracellular (cytosolic to mitochondrial) or cell-to-cell (extracellular) (Brooks, 2000, 2002). Later, Brooks (2009) provided evidence that glycolytic and oxidative pathways should be viewed as linked, as opposed to alternative processes, because lactate, the product of one pathway, is the substrate for the other (Brooks, 2009). The concept of intracellular lactate shuttles was challenged (Sahlin et al., 2002), and continues to be so (Laureys et al., 2014). A study by Cruz et al. (2012) discussed these challenges but ultimately reiterated Brooks' sentiment regarding interaction between energy systems—the product of one is the substrate of another. Soon after Brooks first introduced the concept of lactate shuttles in 1986, several studies by Schurr et al. (1988, 1997a,b,c, 1999a,b) provided experimental evidence that astrocytic lactate is even used preferentially over glucose by neurons after episodes of cerebral ischemia.

Thus, lactate plays an important role as a shuttle, even beyond the brain—in numerous systems, for example, in pregnancy (Zuo et al., 2015), in reproduction (Kuchiiwa et al., 2011), and, notably, within the human heart (Cruz et al., 2012; Rakus et al., 2016). This paper provides an overview of brain neuroenergetics and the crucial roles of lactate shuttles. These roles will be discussed: (1) with regard to the normal physiological function and relationship between astrocytes and neurons (homeostasis)—the astrocyte–neuron lactate shuttle (ANLS) model; (2) the dynamic role of microglia and their preferential utilization of lactate under perturbed conditions (allostasis); (3) the activity of lactate and its shuttles in neuropathological states (in particular in traumatic brain injury and neurodegenerative diseases); and (4) the brain in crisis—in response to a neuroinflammatory infectious disease—according to the astrocyte–microglia lactate shuttle (AMLS) model, using tuberculous meningitis (TBM) as an example.

## Homeostatic neuroenergetics

### Shifting neuroenergetics paradigms

The classical view of neuroenergetics is that the blood supplies oxygen and glucose to the brain (Sokoloff, 1989). Glucose is the primary source of energy utilized by both neurons and astrocytes. It undergoes complete oxidation via glycolysis, the Krebs cycle and oxidative phosphorylation, which ultimately produces adenosine triphosphate (ATP) for energy-dependent reactions. Thus, glucose is used in the same way by all cell types. Since neurons consume the greatest quantity of energy of all brain elements, metabolic intermediates (e.g., in the Krebs cycle) are diverted toward neurons. Some of the pyruvate produced by glycolysis is converted to lactate and released into the extracellular space. In this classical view, lactate is considered a by-product with deleterious effects when in excess (Norenberg et al., 1987; Siesjö, 1988; Bender et al., 1997). Astrocytes have long been thought to play a passive role in supporting neuronal function, with the neuron being the star of the show. However, the dynamic involvement of astrocytes (Ranjbar and Amiri, 2015) in the forefront of neuroenergetics is now being recognized, shifting paradigms (Haydon and Carmignoto, 2006; Giaume et al., 2010). The neurocentric view of neuroenergetics is evolving into a more integrated one of complementary and co-operative metabolic interactions between astrocytes and neurons.

### Astrocytes–more than a supporting role?

At the cellular level the human brain consists of the all-important neurons and up to 10 times more glial cells than neurons (Kimelberg and Norenberg, 1989). There are four types of glial cells: ependymal cells, oligodendrocytes, astrocytes, and microglia (resident macrophages in the brain). Astrocytes constitute about 50% of the total human brain volume and are classically divided into three types based on morphology and spatial organization. These three types are: (1) radial—orientated perpendicular to ventricular surfaces with long, unbranched processes (end-feet); (2) protoplasmic—displaying bushy morphology with numerous, highly branched, short processes; and (3) fibrous—manifesting stellate shapes with smooth, long processes that are less branched. The cytoarchitectural organization of astrocytes is such that, according to Pellerin (2010) particular sections cover 99% of the surface area of cerebral blood vessels; although, due to tissue shrinkage with chemical fixation (Korogod et al., 2015), this 99% value is likely an inflated estimate. These astrocytes are, however, the preferential site for glucose uptake from the blood, as well as having projections in peri-synaptic areas of neurons, providing close interaction with neuronal elements and acting as a cellular barrier between blood and neurons. The unique morphological and phenotypic characteristics of astrocytes ideally position them to sense and respond dynamically to changes in neuronal activity (Pellerin, 2010; Bélanger et al., 2011), lending them to conduct numerous critical functions (Chen and Swanson, 2003; Steele and Robinson, 2012), such as glutamate homeostasis (in the glutamate–glutamine cycle), maintaining brain ionic equilibrium (K^+^ and H^+^ buffering), the maintenance of reactive oxygen species (ROS) (in glutathione recycling) and osmotic regulation. Astrocytes therefore support neuronal activity via structural, trophic and metabolic means, suggesting a critical role in regulating neuroenergetics and homeostatic functions (Pellerin, 2010). Notably, neurons rely on astrocytes to supply precursors of the Krebs cycle intermediates, or their derivatives, as the enzyme pyruvate carboxylase is present in only astrocytes but not in neurons (Hertz et al., 1999).

Astrocytes exhibit a higher capacity for glucose utilization, as well as greater metabolic plasticity, than neurons; these characteristics are important for homeostatic and neuroprotective functions. The calculated energy needs of astrocytes only represent about 10–15% of the total brain energy needs (Attwell and Laughlin, 2001; Gjedde et al., 2002; Rothman et al., 2003; Shulman et al., 2004). Hence, approximately 85% of the glucose in the brain is used in the expenditure of energy in neurons via the glycolytic pathway and the Krebs cycle leading to the synthesis of ATP (Jueptner and Weiller, 1995; Attwell and Laughlin, 2001). The high glycolytic rate of astrocytes suggests a preference for the production and release of lactate. The neuroprotective role of lactate has been experimentally demonstrated by studies (Cater et al., 2001, 2003). Neuroprotection here is defined as an intervention that prevents the death of vulnerable neurons and slows disease progression. Hence, evidence that has emerged over the past two decades has begun to highlight lactate as a supplementary substrate for neurons, resulting in the (re)emergence of a dynamic nursing role for astrocytes (Bouzier-Sore et al., 2002).

### Astrocyte–neuron lactate shuttle (ANLS) hypothesis

**Figure 1:**
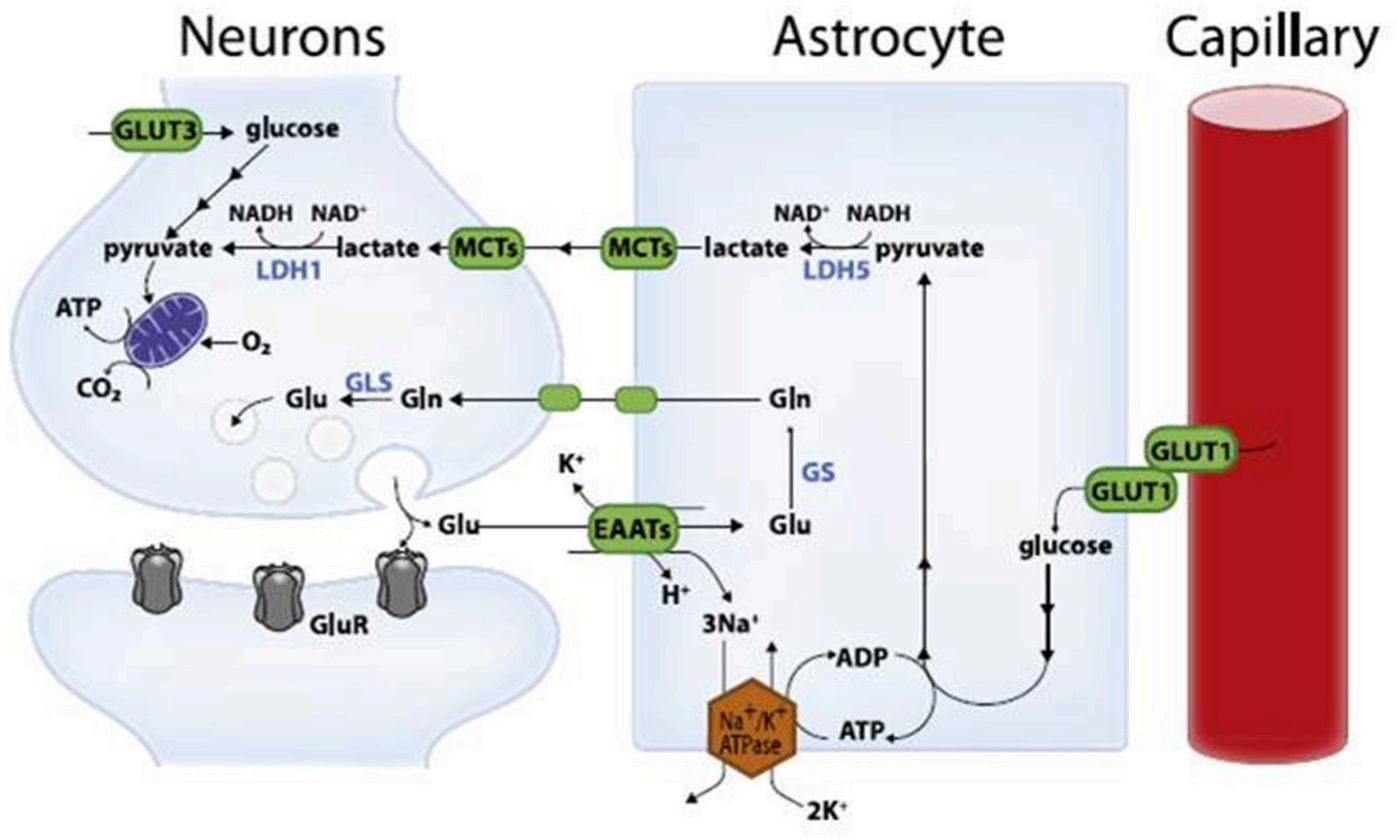
**Schematic representation of the astrocyte-neuron lactate shuttle model (with permission from Bélanger et al., 2011)**. Glu, glutamate; Gln, glutamine; GluR, glutamatergic receptor; EAATs, excitatory amino acid transporters; GLUT, glucose transporter; MCTs, monocarboxylate transporters; LDH, lactate dehydrogenase; GS, glutaminase; GLS, glutamine synthetase.

Magistretti and Pellerin (1996) presented the framework of a hypothesis that they have since developed and refined to become one of the prevailing contemporary viewpoints of neuroenergetics—the ANLS hypothesis. The hypothesis states that astrocytes respond to intensified neuron activity by increasing their rate of glucose uptake, glycolysis and the release of lactate into the extracellular space, as shown schematically in Figure 1. At the metabolic level, it begins with glutamatergic activity, a process whereby increased neuronal activity results in the release of glutamate, the main excitatory neurotransmitter in the brain, into the extracellular space along the glutamate transporter EAAT3, which is exclusively located in neurons. Astrocytes sense increased activity at the glutamatergic synapses, followed by glutamate uptake via the glia-specific glutamate transporters EAAT1 and EAAT2. The transport of glutamate is driven by a sodium gradient (i.e., by a Na^+^-dependent mechanism), with a stoichiometry of three Na^+^ ions co-transported with one glutamate, resulting in a significant increase in intracellular Na^+^ concentrations in astrocytes (Magistretti and Pellerin, 1999; Pellerin and Magistretti, 2004; Bélanger et al., 2011). Glutamate taken up by astrocytes is converted to glutamine through an ATP-dependent reaction catalyzed by astrocyte-specific glutamine synthetase. Glutamine is released back into the extracellular space and taken up by neurons, where it is converted to glutamate by glutaminase (Magistretti and Pellerin, 1999; Bélanger et al., 2011). This reaction thereby replenishes the neurotransmitter pool of glutamate and completes the glutamate–glutamine cycle. Glutamate uptake by astrocytes stimulates glucose uptake with a stoichiometric relationship of 1:1 between uptake of glutamate and glucose. Increased concentrations of Na^+^ in astrocytes activate the enzyme Na^+^-K^+^-ATPase, particularly the α_2_ subunit. The result is the triggering of glycolysis, leading to the production and release of lactate into the extracellular space; the lactate is then taken up as an energy substrate by neurons for oxidative-derived ATP production (Debernardi et al., 1999; Magistretti and Pellerin, 1999; Pellerin, 2003; Pellerin and Magistretti, 2004; Pellerin et al., 2007; Bélanger et al., 2011). This demonstrates the presence of open astroglial metabolic networks—an intercellular route, allowing the trafficking of energy substrates through astrocytes from their source, which is blood vessels, to the site of high energy demand and use, the neurons (Giaume et al., 2010).

Fox et al. (1988) were the first to show that focal, physiological increase in neuronal activity induced by visual stimulation in humans was associated with increased glucose uptake and blood flow in the human visual cortex. This suggested that increased neuronal functional activity stimulates glycolysis. Prichard et al. (1991) and Sappey-Marinier et al. (1992) further developed this postulate by demonstrating increased levels of lactate in the human visual cortex using nuclear magnetic resonance (NMR) technology, following visual stimulation. Thus, a tight coupling was demonstrated between neuronal activation and glucose utilization in astrocytes (Magistretti, 2006), an intrinsic feature of astrocytes not specifically linked to culture conditions or cell origin (Pellerin et al., 2007). It is interesting to note that glucose uptake by astrocytes is disproportionately high compared to their energy requirements. This suggests that sustained astrocytic glycolysis occurs in order to maintain the extracellular lactate pool to meet the energy requirements of neurons. Most recently, data from Angamo et al. (2016) suggest that astrocytic neuronal lactate shuttles contribute to the regulation of ion homeostasis and synaptic signaling in the presence of ample glucose.

In addition to blood-borne glucose, the brain also makes use of an important energy reserve in the form of glycogen stores located almost exclusively in astrocytes. Mobilization of glycogen occurs without ATP requirement during extended periods of limited energy supply (e.g., in hypoglycemia) and leads to enhanced lactate production and release, thereby maintaining energetic homeostasis and preserving neuronal function and viability (Wender et al., 2000; Pellerin, 2003; Pellerin and Magistretti, 2004). Glycogen mobilization can thus be viewed as an extension of the ANLS concept instead of a competing hypothesis (Pellerin et al., 2007). But, as stated by Dienel and Cruz (2015), glycogen is very difficult to study in the human brain. Roles for glycogen in brain function continue to emerge, revealing increasing complexity of the signaling and regulatory mechanisms that integrate glycogen mobilization with physiological activities.

It is important to note that the ANLS hypothesis does not preclude glucose as a source of energy for the brain. Glucose remains an important energy substrate with concentration gradients facilitating the transport of glucose across the blood–brain barrier into the brain via glucose transporters (GLUTs). This gradient exists since concentrations of glucose in plasma and CSF are approximately 5 mM (Psychogios et al., 2011) and 3 mM (Wishart et al., 2008) respectively, with a CSF:plasma glucose ratio typically being approximately 0.4–0.8 (Leen et al., 2012). GLUT1 is specific to astrocytes and cerebral blood vessels, GLUT3 is exclusively localized at neurons, and GLUT5, primarily a fructose transporter, is found in microglia (Vannucci et al., 1997).

Other important regulating proteins in neuroenergetics are: pyruvate dehydrogenase, the rate-limiting enzyme that catalyzes lactate oxidation in astrocytes, with the inactive form being greater in astrocytes than in neurons (Itoh et al., 2003); lactate dehydrogenase (LDH), of which isoenzymes are found to be specific to astrocytes (LDH–5: associated with glycolysis) and neurons (LDH–1: associated with oxidative metabolism) (Bittar et al., 1996; Laughton et al., 2000); and monocarboxylate transporters (MCTs), proton-linked transporters of lactate with localization of subtypes MCT2 to neurons (with high affinity for lactate) and MCT1 and MCT4 to blood vessels and astrocytes, respectively (with lower affinity for lactate) (Bergersen, 2007). The kinetics of these regulating proteins supports the concept of a flux of lactate from astrocyte to neuron. Most recently in literature there has been renewed vigor in the research in the role of MCTs (Bourgeois et al., 2016; Chaumeil et al., 2016; Karagiannis et al., 2016; Kolko et al., 2016; Lee and Kang, 2016; Pérez-Escuredo et al., 2016; Rosafio et al., 2016). Hence, we are entering an era in science whereby we are recognizing that understanding not only shuttles, but the means by which shuttles operate—transporters (e.g., MCTs), are integral toward unraveling new information of the complex dynamic processes found in neuroenergetics.

Astrocytes are therefore viewed as “lactate sources” supplying the extracellular lactate pool, and neurons as “lactate sinks” consuming lactate oxidatively in response to energy demands. The transfer of lactate from astrocyte to neuron can be viewed as a spatially and temporally independent process (Pellerin and Magistretti, 2004). Thus, the metabolic plasticity of astrocytes is likely to be associated with synaptic plasticity (Magistretti, 2006). Magistretti (2000, 2009) sums up the ANLS model in terms of neurometabolic coupling in which sodium-coupled uptake of glutamate by astrocytes activates Na^+^-K^+^-ATPase, which triggers glucose uptake and its glycolytic processing. This results in the production and release of lactate that is used by neurons for activity-dependent energy demands. It has become generally accepted that lactate is a pivotal component in neuronal brain energy homeostasis (Schurr et al., 1999a; Pellerin, 2003, 2010; Gladden, 2004; Schurr, 2006; van Hall et al., 2009).

### Controversy surrounding the ANLS hypothesis–is this the new paradigm?

Since its introduction, the ANLS has received much attention, some of which criticizes and refutes the concept; however, substantial evidence has accumulated from independent sources that supports the model. Based on *in vitro* and *in vivo* studies (Kuhr and Korf, 1988; Pellerin et al., 2002, 2007; Itoh et al., 2003; Smith et al., 2003; Pellerin and Magistretti, 2004; Hashimoto et al., 2008; Sampol et al., 2013), there exists experimental evidence that neurons use lactate derived from astroglial metabolism efficiently as energy substrates to preserve normal neuronal function. The lactate shuttle concept at the core of the ANLS was further supported by studies on the critical role of glutamate transporters (Pierre et al., 2000; Voutsinos-Porche et al., 2003) and distribution of MCTs in the brain (Pierre et al., 2000; Chiry et al., 2008; Erlichman et al., 2008; Robinet and Pellerin, 2011), particularly for long-term memory formation (Suzuki et al., 2011).

Molecular mechanisms provide further *in vitro* evidence that supports the ANLS hypothesis (Bliss et al., 2004; Porras et al., 2004), showing that upregulation of glycolysis in neurons decreased oxidation of glucose through the pentose phosphate pathway, resulting in impaired regeneration of reduced glutathione, and subsequently oxidative stress and apoptotic death. Thus, neurons downregulate glycolysis in order to use available glucose to maintain antioxidant status (a neuroprotective mechanism) at the expense of its use for bioenergetics purposes. Neuroenergetic demands can be met by other sources, such as lactate (Tabernero et al., 1996; Herrero-Mendez et al., 2009).

Rouach et al. (2008) demonstrated *in vitro* the change in character of astrocyte metabolic networks in response to local energy demand and trafficking of energetic metabolites from blood vessels, through astrocytes, to distal neurons. Using ^13^C-NMR, this metabolic flux of lactate from astrocyte to neuron can be measured noninvasively in the human brain (Bouzier-Sore et al., 2003; Gallagher et al., 2009; Boumezbeur et al., 2010). Plasma lactate supports up to 10% of brain energy metabolism under physiological conditions, and up to 60% under supra-physiological conditions. Mathematical modeling studies have also described these dynamics, in particular brain lactate kinetics (Aubert et al., 2005) and compartmentalization of brain energy metabolism (Aubert and Costalat, 2005). However, as stated by Pellerin et al. (2007): “[mathematical modeling] …can give a coherent, quantitative framework for the discussion, suggest possible mechanisms or, conversely, emphasize the contradictions or implausibility or some hypotheses.” An example of the argument about the possible ambiguity of mathematical modeling is given by Genc et al. (2011). This sentiment is further expressed by Jolivet et al. (2010): “mathematical models are very powerful tools but they are ultimately only partial replicas of the system they model, not the system itself.” Thus, mathematical models are useful guides but one should be mindful to draw definitive conclusions based on modeling studies alone. Jolivet et al. (2010) commented on two mathematical studies of hypotheses of opposing lactate flux in neuroenergetics—they compared the mathematical model of ANLS by Aubert et al. (2007) and the neuron-to-astrocyte lactate shuttle (NALS) by Mangia et al. (2009). Jolivet et al. (2010) concluded that the Aubert (ANLS) model currently remains the best biophysical representation of neuron–astrocyte metabolic interactions. Further multi-timescale mathematical modeling by Jolivet et al. (2015) has since provided further support for the ANLS hypothesis.

While less vocal, there remains strong opposition to the ANLS hypothesis, with various studies expressing divergent views. Dienel and Hertz (2001) emphasized the paradox of intense production of lactate by the brain and the slow rate of its uptake, thus disagreeing that lactate is used as a major neuronal fuel. Dienel and Cruz (2003, 2004) further argued that cerebral metabolic rates of oxygen consumption do not equal that of glucose plus glycogen and that this disproportionate consumption is strong evidence against stoichiometric transfer of lactate from astrocytes to neighboring neurons for oxidation. Gjedde et al. (2002) averred that, based on their experimental evidence, changes in metabolism in afferent phase (activity involving presynaptic terminals and astrocytes) and efferent phase (activity involving neurons) are additive. This view opposed the idea that changes in metabolism are characterized by significant transfer of lactate from astrocyte to neurons. Gjedde et al. (2002) conclude that there is no suggestion that astrocytic glycolysis supports oxidative metabolism of neurons in the baseline condition. Instead, neurons increase their oxidative metabolism in parallel with a rise in pyruvate generated by neuronal rather than astrocytic glycolysis (Gjedde and Marrett, 2001). Mangia et al. (2003a) discussed various ambiguities from other studies and conclude that astrocyte activation supports only the glutamate–glutamine cycle and that neurons primarily metabolize directly absorbed glucose to support neuronal activity. This behavior is demonstrated by an initial dip in lactate concentrations following visual stimulation (Mangia et al., 2003b). As mentioned above, Mangia et al. (2009) also proposed a model of opposite flux to the ANLS, namely the NALS hypothesis which utilized the mathematical model introduced by Simpson et al. (2007), suggesting that depending on the thermodynamic and kinetic status of the cytosolic and mitochondrial redox states, lactate transfers from neurons to astrocytes. The directionality of the ANLS hypothesis (i.e., lactate from astrocytes to neurons), has been covered in many reviews. However, the other perspective of shuttling direction (i.e., from neurons to astrocytes) is an intriguing one, albeit without substantiated experimental proof of the NALS, beyond modeling. Other divergent views come from studies by, for example, Bak et al. (2006), who suggested that synaptic activity does not induce corresponding upregulation of lactate metabolism in neurons; DiNuzzo et al. (2010a), who suggested that carbon recruitment by neurons relies upon glucose uptake rather than that of a lactate shuttle, and that glycogenolysis in astrocytes preserves glucose availability for neurons (DiNuzzo et al., 2010b).

Others have critically reviewed the ANLS hypothesis in defense of the traditional role of neuroenergetics (Chih et al., 2001; Chih and Roberts, 2003). Pellerin and Magistretti (2011) not only addressed all these divergent views by reviewing experimental evidence supporting the main tenets of the ANLS model, but also were able to confute several unfounded criticisms. With overwhelming support for the ANLS hypothesis, it was becoming clear that lactate is a crucial element in neuronal brain energy homeostasis (Schurr et al., 1999b; Pellerin, 2003, 2010; Gladden, 2004; Schurr, 2006; van Hall et al., 2009). However, as is the case in science, refuting evidence continues to challenge the foundations of the ANLS hypothesis, leading to it being redefined and strengthened over time. The tenets of this hypothesis continue to hold, begging the question—is this the new paradigm for homeostatic neuroenergetics?

## Neuropathology and the human response

### Beyond homeostasis

Biological processes maintain stability, by detecting environmental (external) and physiological (internal) changes, and activating specialized adaptive responses. The dynamic metabolic characteristics of astrocytes lend them to being particularly adept at such a task within the neuronal framework of homeostasis. Beyond homeostasis lies the comparatively new concept of allostasis. Allostasis is an extension of the concept of homeostasis and refers to the maintenance of stability by means of robust, energy-demanding adaptive mechanisms in response to severe physical, psychosocial or environmental challenges. Homeostasis involves maintaining a *stable* internal environment of an organism (i.e., by means of a *feedback* mechanism), whereas allostasis is more *dynamic* in that it involves the continuous response to physiological needs, resulting in biochemical *adaptive* mechanisms. These two concepts seem similar and are not intended to operate independently; instead, allostasis supports homeostasis, placing emphasis on flexible adaptation with the ultimate goal of maintaining a stable internal environment (McEwen, 2006, 2008; Logan and Barksdale, 2008; McEwen and Gianaros, 2011; Danese and McEwen, 2012). Frequent or chronic challenges (e.g., neuroinflammation) produce dysregulation of several major physiological systems by triggering chemical mediators of adaption that operate in a nonlinear network. The cumulative “wear and tear” associated with the inability to disengage these physiological systems is referred to as allostatic load. In vulnerable biological systems an allostatic overload prevails that results in the development of disease.

Since the term was first coined, by Sterling and Eyer (1988), the concept of allostasis has primarily been used in the literature to describe mild perturbations that are stress related, psychosomatic and/or psychopathological (Tannenbaum et al., 2002; Stewart, 2006; Shannon et al., 2007; Logan and Barksdale, 2008; Blair et al., 2011; Danese and McEwen, 2012; Tomiyama et al., 2012). As a concept allostasis is still being developed and so is gradually being applied to more diverse fields, such as metabolic diseases and lipidomics (Oresic et al., 2008) and metabolomics (Ramautar et al., 2013). Allostasis has also been used to explain extreme glucose fluctuations as reflecting the body's inability to cope with allostatic load in the case of chronic illness, predisposing the individual to serious harm as manifested by heightened mortality (Stumvoll et al., 2003, 2004; Rake et al., 2010).

Herein this review the typology, as given by Peters and McEwen (2015) to describe cardiovascular disease, has been adapted to define terms in neuroenergetics. Stress occurs in a state of increased cerebral energy demand to safeguard an individual's physical, mental and social well-being. A reversible stress response distinguishes allostasis from homeostasis and can be indicated by a relief result (restored homeostasis) in which the individual returns toward physiological normality. An allostatic load is indicated by an acute stress challenge—an irreversible or nearly irreversible state of allostatic stress from homeostasis—as seen in severely elevated CSF lactate in acute, physical damage to the brain (Gallagher et al., 2009). Chronic cases of persistently highly elevated CSF lactate and lactate:pyruvate ratios would be parameters to define the transition of allostatic load toward allostatic overload (disease).

Thus, allostasis encompasses typical dynamic adaptations to transient stress mediators; allostatic load pertains to an acute state of persistent perturbation and, ultimately, circumstances of allostatic overload are associated with disease and the body's inability to cope. These terms are henceforth discussed in terms of neuropathological conditions, with the focus on lactate and its shuttles.

### Microglia–addressing neuronal allostasis

Under pathological conditions, such as caused by infection, the brain enters a state of allostasis and microglia become activated. Microglia are immunocompetent cells that act as the intrinsic macrophages of the brain, bearing multiple similarities to those of macrophages in peripheral tissues. Some of the shared functions include phagocytosis, antigen presentation, effector inflammatory response and production of various cytotoxins and cytokines. Thus, microglia are an important component of both the innate and adaptive immune response to central nervous system (CNS) pathogens (Olson and Miller, 2004). They populate the entire brain parenchyma with a homogeneous distributed network with territorial organization and act as the first and only line of defense in the brain.

Under physiological conditions microglia exist in a resting (“ramified”) state characterized by a small soma and numerous thin, branched processes (Giaume et al., 2007). Microglia remain quiescent until activated upon by brain insult (i.e., injury, disease or infection), characterized by proliferation and immunophenotypical (expression of various surface markers and ion channels) and morphological (transformation to amoeboid morphology) changes (Eder, 1998). The response of microglia to brain insult is first to detect the site of assault by constant dynamic monitoring of the surrounding micro-environment, and then to send out process extensions toward the lesion site where process tips reorganize to confine, control and eliminate the source of the disturbance.

Neuroinflammation, the reaction of surrounding brain tissue to brain insult, is characterized by synthesis of various inflammatory mediators and reactive gliosis, associated with phenotypic changes and proliferation of glia (both astrocytes and microglia), in response to a dynamically changing environment (Giaume et al., 2007). The modified phenotype of astrocytes, from basal to reactive state, results in them abandoning their neuroprotective role, allowing excessive oxidative stress and production of ROS, and subsequently ROS-induced neuronal damage (Pellerin, 2003). Chronic neuroinflammation, the response to sustained and widespread stimuli, typically occurs due to either disease (e.g., Alzheimer's and multiple sclerosis) or persistent infection by viruses (Olson et al., 2001; Ovanesov et al., 2008) or bacteria (e.g., chronic meningitis as in TBM). The role of microglia in CNS infections was extensively reviewed by Rock et al. (2004). Hence, metabolic coupling of microglia is essential during neuronal allostasis, especially in response to pathogens.

## The brain in crisis

### Allostatic load–physical brain trauma

The topic of traumatic brain injury (TBI)—acute, physical damage to the brain—gained notoriety in 2009 when Jeanne Marie Laskas wrote an article titled “Game brain” in GQ magazine (Laskas, 2009), exposing the hidden, negative medical implications within some popular, professional contact sports. In 2015 this was expanded into a book (Laskas, 2015) and a film called “Concussion,” about forensic pathologist Bennet Omalu and his attempts to publicize his scientific findings regarding physical contact sports (such as in the National Football League, the apex of American football). Omalu postulated that persistent mild TBI leads to chronic traumatic encephalopathy. Consequently, there has been renewed research into concussion and TBI (Sone et al., 2016; Sussman et al., 2016), making it another hot topic in neuroscience.

It is well recognized that there is an elevation of brain extracellular lactate and the lactate:pyruvate ratio in TBI cases (Gallagher et al., 2009). The group of Gallagher et al. (2009) and Carpenter et al. (2014) presented direct evidence of brain utilization of lactate in TBI cases by administration of ^13^C-labeled lactate via a microdialysis catheter and using ^13^C-NMR analysis. Based on these studies, Carpenter et al. (2015) suggested that where neurons are too damaged to use the lactate produced from glucose by astrocytes (i.e., by uncoupling of neuronal and glial metabolism), high extracellular levels of lactate would accumulate—explaining the association between high lactate and poor outcome. Thus, therapeutic intervention before metabolic uncoupling is imperative. Glucose administration after TBI has been shown to be beneficial but there is a correlation between hyperglycemia and increased infection or mortality; hence, alternative sources of energy, such as lactate, may improve outcome (Moro et al., 2013). However, as with the ANLS model, there are opposing opinions of the notion that lactate is a preferential fuel in TBI cases (Dienel, 2014).

Acute administration of exogenous fuels, such as lactate, after experimentally induced TBI in rats has been shown to attenuate histopathology and improve outcome (Chen et al., 2000). In two companion reports by Glenn et al. (2015a,b), evidence was provided that central venous tracer infusion of both glucose and lactate showed massive mobilization of mainly lactate—systematic lactate was preferentially being directly consumed and used by TBI cases. Thus, there is a high production and clearance rate of lactate in TBI cases. The role of this lactate in cerebral metabolism following TBI and the advantages of treatment by exogenous lactate infusions was reviewed by Brooks and Martin (2015). In recent clinical trials of TBI, and other neurocritical care cases, the beneficial properties of hypertonic lactate have been observed with respect to cerebral blood flow and intracranial pressure (Bouzat and Oddo, 2014; Patet et al., 2016). Thus, accumulating evidence points to lactate being an important component used during allostatic load caused by acute, physical damage to the brain—a therapeutic target.

### Allostatic overload–neurodegenerative diseases

A common trait among neurodegenerative diseases is perturbed brain energy metabolism (Magistretti and Pellerin, 1996; Beal, 2000; Bélanger et al., 2011; Albanese et al., 2016). Huntington's disease and multiple sclerosis have been linked to mitochondrial dysfunction, and so are susceptible to oxidative stress and energy deficits (Dutta et al., 2006; Regenold et al., 2008; Sack, 2010; Gouarné et al., 2013); with implications of perturbed lactate levels. In contradiction, two ^1^H-NMR studies found conflicting results regarding lactate in Huntington's disease. An ^1^H-NMR investigation by Gårseth et al. (2000) reported decreased levels of lactate, which they attributed to neuronal loss, but based upon only a small sample (*n* = 7). Another ^1^H-NMR study, by Verwaest et al. (2011), found lactate to be significantly increased, but this result was also based on a small sample (*n* = 10). In a more comprehensive study, Gouarné et al. (2013) used cultured neuronal subpopulations from transgenic mice and provided evidence that neurons use lactate, along with pyruvate, as an energy source to support respiration. The importance of lactate was corroborated by a study (Covarrubias-Pinto et al., 2015) on Huntington's disease (HD) using ascorbic acid to inhibit use of neuronal glucose—showing the favoring of lactate uptake to sustain brain activity. Hence, experimental evidence is emerging that lactate is important and used preferentially as an energy source in HD cases.

Two reviews on neurodegenerative diseases support the ANLS hypothesis and its role in neuropathology—namely, lactate is neuroprotective and a therapeutic agent. A review by Newington et al. (2013) refers to their previous scientific work, which showed that increased lactate production proved to be protective against Aβ-induced neuronal toxicity that is inherently associated with Alzheimer's disease. A review by Finsterwald et al. (2015) summarizes numerous studies involving astrocytic lactate in treatments for multiple neurodegenerative diseases [Alzheimer's, Parkinson's disease and amyotrophic lateral sclerosis (ALS)]. Finsterwald et al. (2015) concluded that astrocytic function elicits intrinsic neuroprotective properties through, amongst others, the ANLS mechanism. One could go one step further and speculate that a “specialized” type of ANLS mechanism is necessary.

In multiple sclerosis (MS) there have been reports of the importance of CSF lactate. A negative correlation between the presence of lactate and the presence of inflammatory plaques and MS severity has been experimentally shown (Lutz et al., 2007; Albanese et al., 2016)—increased lactate in MS cases without plaques and vice versa, suggesting close metabolic coupling between plaque activity and lactate production. A gene expression study by Zeis et al. (2015) revealed alterations (downregulation) in the ANLS mechanism in MS, such that alternative lactate shuttle systems may be at play. Indeed, other studies have proposed such shuttle systems to supply demyelinated axons with lactate as an important energy source—namely, the astrocyte-axon lactate shuttle (Cambron et al., 2012; Nijland et al., 2015) and the oligodendrocyte-axon lactate shuttle (Campbell et al., 2014).

A common thread seen in these studies of neurodegenerative diseases is the perturbation of lactate. Evidence in neuropathophysiological cases indicates that lactate acts as an important, sometimes preferential, source of energy, and also has neuroprotective properties. The role of lactate shuttle systems is also emerging as being vital—imperative as supply and demand needs fluctuate to extremes in situations of allostatic overload. This is when normal physiological systems can no longer provide the required resources and/or effects. It is, however, important to note that neuropathologically activated astrocytes, as well as other glial cells, such as microglia, may support mechanisms, such as neuroinflammation, aggravating neuronal degeneration (Li et al., 2011). The dynamics behind addressing neuroinflammation caused by an invading pathogen involves the use of microglia.

In the remainder of this review an adaptation of the ANLS model—the AMLS model (Mason et al., 2015)—is discussed in terms of TBM; a chronic, infectious neuroinflammatory disease.

### Pathogen-induced chronic neuroinflammation

**Figure 2:**
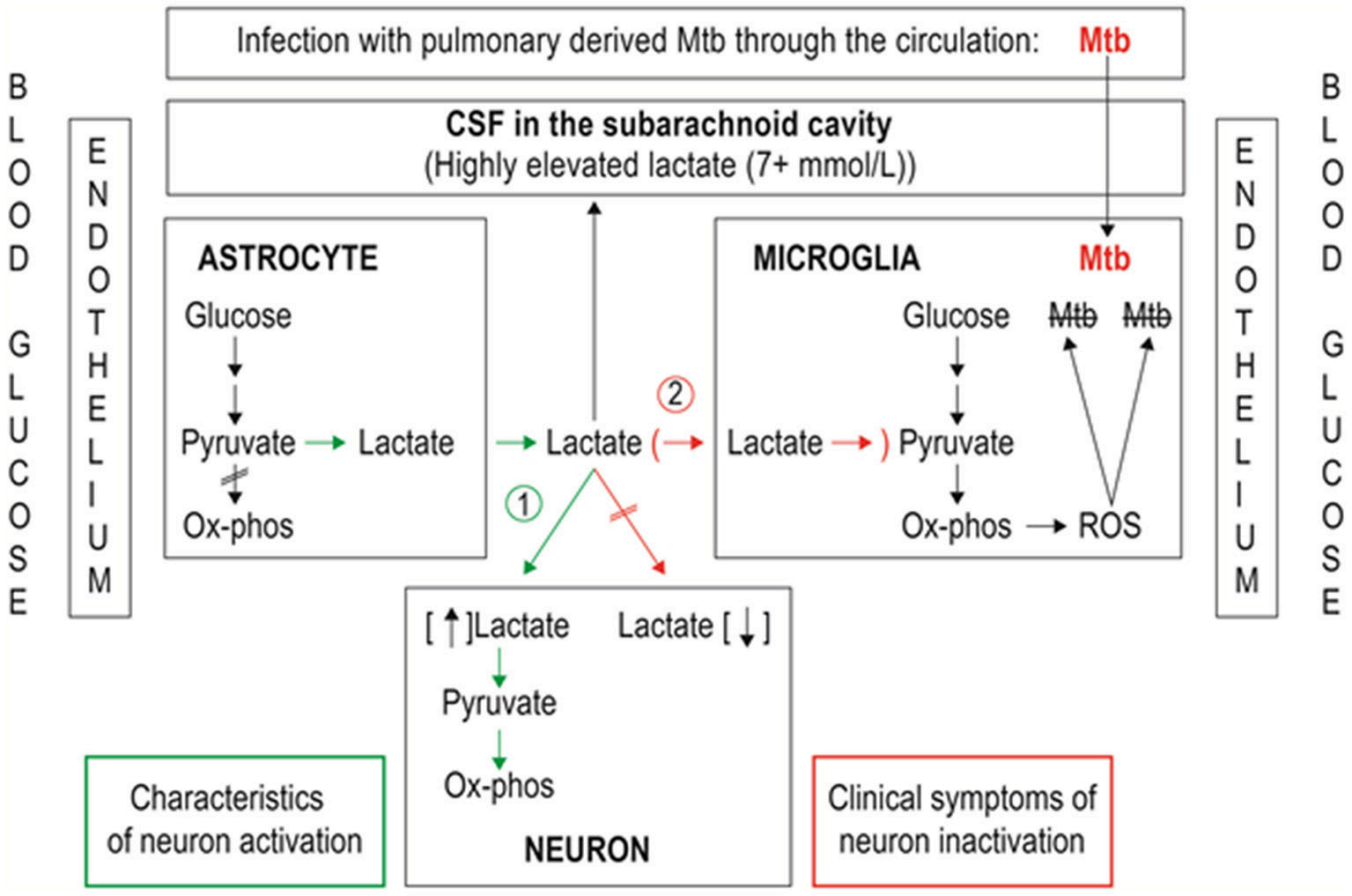
**Representation of metabolic pathways of two lactate shuttles within the central nervous system**. (1) The astrocyte–neuron lactate shuttle (ANLS; green pathway) operates under normal physiological conditions with astrocytes responding to glutamatergic activation by increasing their rate of glucose utilization and release of lactate in the extracellular space, making the lactate available for neurons to sustain their energy demands. (2) The astrocyte–microglia lactate shuttle (AMLS; red pathway) proposed for tuberculous meningitis (TBM). Astrocytes respond to signaling from *Mycobacterium tuberculosis* (Mtb)-infected microglia by increasing glucose mobilization, leading to increased extracellular lactate, reflected in increased cerebrospinal fluid (CSF) lactate levels. Lactate used by microglia as an additional energy source for reactive oxygen species (ROS) production aimed at destroying the invading Mtb.

In the case of a chronic infection in the brain by a persistent pathogen, such as *Mycobacterium tuberculosis* (Mtb), the bacillus responsible for TBM, there is sustained allostatic overload. Here, the microglia strive to eradicate the scourge but unwittingly become the habitat of the persisting pathogen. Similar to the ANLS hypothesis, the AMLS model proposes that when the brain is in crisis due to infection, energy flow in brain metabolism is shifted away from the neurons, and shunted toward the microglia (see Figure 2). The initial mechanics of the AMLS remain the same as the ANLS, the only difference being the directionality of the lactate upon exiting the astrocyte. The AMLS hypothesis postulates that in neuroinflammatory infectious diseases, such as TBM, lactate produced through glycolysis in astrocytes participates in the activated immune response and, in association with ketones and gluconeogenic amino acids, is collectively directed from the neurons preferentially into microglia. Within the microglia, lactate is expected to enter the mitochondrial Krebs cycle, contributing to oxidative phosphorylation and hence producing high levels of ATP and forms of ROS, such as hydrogen peroxide, required for degradation of the invading pathogen. Thus, the AMLS hypothesis uses the same line of logic of the ANLS model, but instead the microglia are the prime focus under conditions of neuroinflammatory infectious diseases; increased astrocytic lactate is directed toward the microglia.

Using the same CSF samples from the study by Mason et al. (2015), it was shown in a targeted analysis (Mason et al., 2016a) that the highly elevated amounts of lactate observed in the CSF of confirmed TBM cases was only in the L-form (L-lactate is produced by the host, whereas D-lactate is of bacterial origin). Thus, elevated lactate found in the CSF of TBM cases is solely a response by the host to the infection. These results from this follow-up study by Mason et al. (2016a) provide experimental evidence to support the proposed AMLS model and highlight the fact that lactate plays an important role in neuroinflammatory diseases, such as TBM. Activation of microglia, as required in the AMLS hypothesis, does not, however, present a uniform process and involves intricate interactions and feedback loops between the microglia, astrocytes and neurons that hamper attempts to construct basic and linear cascades of cause and effect; TBM involves a complex integration of the responses from the various cell types present within the CNS, with microglia and the astrocytes as main players.

During the course of a neuroinflammatory infectious disease, such as TBM, the ANLS is not only inadequate but, indeed, a liability as activated neurons will only become vulnerable to neurodestructive components. The bypassing of metabolic energy intermediates from the neurons effectively inactivates them to protect them from neurodestructive agents in response to chronic neuroinflammation. This neuron inactivation is evident from a clinical viewpoint as the progression of TBM is reflected by reduced scoring in the Glasgow Coma Score—specifically, decreased consciousness and awareness. The AMLS hypothesis can thus be viewed as invoking a persistent allostatic overload state that becomes activated when allostatic mechanisms fail and the body enters into a diseased state. The regulation of dynamic systems that function within the brain, in terms of homeostasis, allostasis and disease states, is delicate. Chronic activation of microglia can lead to neuropathological sequelae (Streit et al., 2004), and is also linked to neuropathic pain (Raghavendra et al., 2003). The persistent activation of microglia can be viewed as the proverbial double-edged sword—simultaneously exhibiting neuroprotective and neurodestructive properties in an attempt to save the whole at the expense of the part (Hall et al., 1998; Giaume et al., 2007).

Using TBM as an example of an infectious neuroinflammatory disease—an extreme scenario of allostatic overload—it can be speculated that the Mtb-induced host and microbe markers can reflect: (1) the disease state on admission to hospital, and (2) differentiation in the restoration toward a new condition of homeostasis following treatment. Upon admission to hospital, most TBM patients present with moderate to severe symptoms of ketosis. Metabolomics analysis of the urine (Mason et al., 2016b) substantiates this clinical picture through a ketosis urinary biosignature—cases admitted to hospital with very high ketosis biomarkers tended to have a poor prognosis, leading to severe neurological complications irrespective of treatment. A similar profile was also reflected in the CSF biochemistry (Mason et al., 2016a); as the TBM disease progressed into its later stages there was a deterioration in patient prognosis. Hence, in more advanced TBM stages there is less CSF lactate and therefore fewer energy substrates available for microglia activity, and decreased protection of neurons. During this period, irreversible neurological damage can begin to occur—illustrating the importance of lactate levels during periods of chronic neuroinflammation.

The concept of lactate shuttle systems extends to other types of neuronal cells, such as the oligodendrocyte–axon lactate shuttle (Baltan, 2015) and the Bergmann glia (BG) Purkinje cell (PC) lactate shuttle (Sawada et al., 2016). Lactate shuttles extend beyond the brain—they are of particular interest in cancer research (Pizzuto et al., 2012). Several cancer studies have suggested a tumor-to-stroma coupling via a lactate shuttle in cancer cells (Pértega-Gomes et al., 2014; Sanità et al., 2014). These studies indicate that over-propagation of cells is strongly dependent upon the anaplerotic role of increased lactate uptake (Whitaker-Menezes et al., 2011; Fiaschi et al., 2012). This concept is supported by a large-scale *in silico* metabolic model (Capuani et al., 2015). Interestingly, Somashekar et al. (2011) stated that their most relevant metabolic observations in studying tuberculosis are similar to metabolic changes seen in cancer during tumor development—corroborated by Zhou et al. (2015). Conceptually, the “Reverse Warburg Effect” (Pavlides et al., 2009), proposed as operating in tumor cells, has been interpreted to be analogous to the astrocyte–neuron metabolic coupling (Pavlides et al., 2010), where the astrocytes resemble cancer-associated fibroblasts and the neuron the epithelial tumor cells, resulting in their higher proliferative capacity.

The regulation of lactate shuttles has been discussed as being a potential therapeutic target in cancer research (Draoui and Feron, 2011; Sanità et al., 2014). While disruption of transport of lactate into cancerous cells (i.e., reducing lactate shuttling into cancerous cells) is considered beneficial, upregulation in neuropathological cases shows merit for research. Several studies have demonstrated that intracerebroventricular or intravenous injection of lactate yields a neuroprotective effect during experimentally induced hypoglycemia or cerebral ischemia (Berthet et al., 2009, 2012; Wyss et al., 2011). In addition, other experimental work (Yamanishi et al., 2006; Gordon et al., 2008) has shown that lactate increases cerebral blood flow (vasodilation)—increasing the supply of metabolic (e.g., energy) substrates during allostasis. Thus, lactate has a potentially beneficial role in the clinical management of several neurological disorders (Taher et al., 2016). This review, like the others cited here, therefore shows that further research into the role of lactate and its shuttling mechanism(s) are certainly warranted to address neuroenergetics and neuropathology.

## Concluding remarks

In summary, we are seeing the prevailing school of thought moving away from the neurocentric paradigm, albeit with divergent views, toward that of one that is more integrated. Within this emerging new paradigm, an unraveling of the intricacies of the complex and diverse dynamic interactions of the human brain at the metabolic and cellular levels is occurring. Our current knowledge has led us to the realization that lactate shuttle systems, both intracellular and extracellular, play a far more pivotal role than originally considered. Indeed, there exist numerous other shuttles in neuroenergetics—for example: the lactate–alanine shuttle (Zwingman et al., 2000); the astrocyte–neuron ketone body shuttle (Guzmán and Blázquez, 2001); the glycerol phosphate shuttle (McKenna et al., 2006); and the malate–aspartate shuttle (McKenna et al., 2006; Moffett et al., 2013). Each in its own right is essential in terms of both homeostasis and allostasis. It is evident that transitions between different states of homeostasis and allostasis, and disease, are dynamic—they are often difficult to distinguish and/or control. However, the role of neuroenergetics is undeniable in the brain's constant objective of protecting itself and trying to maintain an optimum state. Here, we have discussed, in terms of neuroenergetics, the role of lactate in a shuttle system during homeostasis (ANLS) and in the infected state (AMLS). Other studies on neurodegenerative diseases have described complex pathophysiological changes that highlighted the importance of lactate (Newington et al., 2013; Finsterwald et al., 2015). There is thus overwhelming evidence that astrocytes, lactate and the associated shuttle systems so far recognized in combination are of crucial importance in neuroenergetics—in both the healthy and diseased state. Perhaps not surprisingly, the latest technology has revealed that neuro(patho)physiological metabolism is far more complex than originally thought and that further knowledge of the lactate shuttle systems apparently operating, as outlined in this article, is key to our understanding of the mechanisms involved.

## Author contributions

The author confirms being the sole contributor of this work and approved it for publication.

## Funding

Research funding was provided by the Technological Innovation Agency (TIA) of the Department of Science and Technology of South Africa. Opinions expressed and conclusions arrived at, are those of the author and are not necessarily to be attributed to the funding body TIA.

### Conflict of interest statement

The author declares that the research was conducted in the absence of any commercial or financial relationships that could be construed as a potential conflict of interest.
